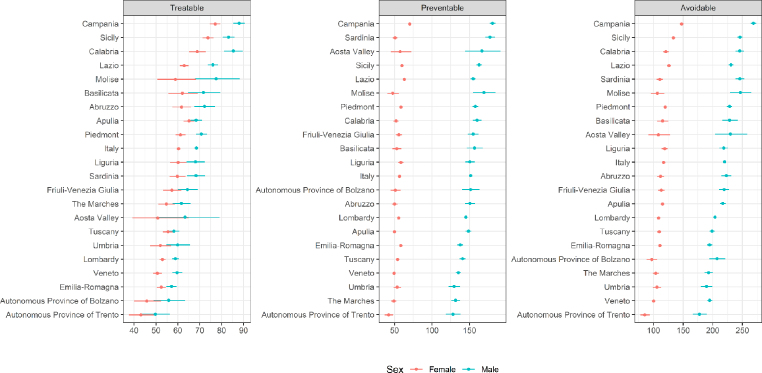# Regional and sex inequalities of avoidable mortality in Italy: A time trend analysis

**DOI:** 10.1016/j.puhip.2023.100449

**Published:** 2023-10-26

**Authors:** Davide Golinelli, Giovanni Guarducci, Andrea Sanna, Jacopo Lenzi, Francesco Sanmarchi, Maria Pia Fantini, Emanuele Montomoli, Nicola Nante

**Affiliations:** a Post Graduate School of Public Health, University of Siena, Italy; b Department of Molecular and Developmental Medicine, University of Siena, Italy; c Department of Biomedical and Neuromotor Sciences, University of Bologna, Italy; d VisMederi S.r.l., Siena, Italy

**Keywords:** Avoidable mortality, Amenable mortality, Treatable mortality, Preventable mortality, Gender differences, Disparities

## Abstract

**Objectives:**

This study provides a comprehensive analysis of avoidable mortality (AM), treatable mortality (TM), and preventable mortality (PM) across Italy, focusing on region- and gender-specific inequalities over a 14-year period.

**Study design:**

Time-trend analysis (2006–2019).

**Methods:**

The study was conducted using mortality data from the Italian Institute of Statistics to evaluate the extent and patterns of AM, TM, and PM in Italy. Biennial age-standardized mortality rates were calculated by gender and region using the joint OECD/Eurostat list.

**Results:**

The overall AM rates showed a large reduction from 2006/7 (221.0 per 100,000) to 2018/9 (166.4 per 100,000). Notably, females consistently displayed lower AM rates than males. Furthermore, both gender differences and the North–South gap of AM decreased during the period studied. The regions with the highest AM rates fluctuated throughout the study period. The highest percentage decrease in AM from 2006/7 to 2018/9, for both males (−41.3 %) and females (−34.2 %), was registered in the autonomous province of Trento, while the lowest reduction was observed in Molise for males (−17.4 %) and in Marche for females (−10.0 %).

**Conclusions:**

Remarkable gender and regional differences in AM between 2006 and 2019 have been recorded in Italy, although they have decreased over years. Continuous monitoring of AM and the implementation of region- and gender-specific interventions is essential to provide valuable insights for both policy and public health practice. This study contributes to the efforts to improve health equity between Italian regions.

## 1 Introduction

### 1.1 Avoidable mortality

The concept of Avoidable Mortality (AM) gained traction as a significant indicator for appraising the quality and efficacy of healthcare systems in recent years [[1], [2], [3]]. Defined by the Organization for Economic Co-operation and Development (OECD) [4,5], AM encapsulates premature deaths (0–74 years) that could be circumvented through the timely and efficacious implementation of healthcare interventions. Such interventions encompass a broad spectrum, including screening, diagnosis, treatment, or vaccination, as well as modifications in lifestyle and environmental factors. The concept of AM is frequently employed as an integrative measure to gauge the quality and effectiveness of healthcare systems, embodying the sum of amenable (or treatable) mortality (TM) and preventable mortality (PM) [[4], [5], [6], [7]]. TM delineates those causes of death that can be primarily circumvented through the timely and effective execution of healthcare interventions. This includes secondary prevention measures and treatments after the onset of diseases aimed at reducing case-fatality rates. Consequently, TM deaths are those that could have been precluded through the efficacious application of healthcare interventions, independent of lifestyle or environmental considerations. Conditions deemed treatable to healthcare interventions typically include certain cancers, cardiovascular diseases, and infectious diseases [8,9]. On the other hand, PM encapsulates deaths that could be forestalled with effective public health and healthcare interventions, which are executed before the onset of diseases or injuries with the aim of reducing incidence rates. Non-treatable conditions, which are not typically responsive to healthcare interventions, such as accidents or suicides, can still be prevented through public health measures like injury prevention programs or mental health initiatives [[1], [2], [3], [4], [5], [6], [7], [8], [9], [10]]

### 1.2 Italian context

The assessment of health system performance has increasing prominence in OECD and EU countries, and Italy, like numerous other nations, grapples with the challenge of curbing AM rates and addressing health disparities. Despite overall advancements in healthcare, Italy continues to confront considerable region- and sex-related differences in health outcomes [[11], [12], [13]]. These disparities can be ascribed to factors such as cultural level, socioeconomic status, access to healthcare, and lifestyle variables.

Like other European countries [11], Italy's inequalities - including sex differences - in AM, TM, and PM are a mounting concern. Women typically exhibit longer life expectancy than men, but concurrently experience distinct mortality patterns, with heightened rates of morbidity and disability. These sex disparities in mortality are propelled by a multitude of social, cultural, and biological factors, with access to healthcare playing a pivotal role in contributing to such differences [12]. Even though women generally demonstrate higher healthcare utilization than men, they encounter hurdles to accessing care, such as transportation difficulties, financial constraints, and cultural norms that discourage care-seeking. As a result, women may not receive prompt and appropriate medical interventions for conditions such as cardiovascular disease or cancer, leading to elevated AM rates. Additionally, lifestyle factors contribute to sex disparities in AM [13]. Italian women, despite lower levels of physical activity, especially after the menopause, typically lead healthier lifestyles, including by lower smoking and alcohol consumption rates. Nonetheless, they may face amplified exposure to environmental toxins, such as indoor air pollution, culminating in increased rates of respiratory disease and other avoidable conditions [[12], [13], [14]].

The primary objective of this study is to analyze the region- and sex-specific trends of AM in Italy, and to provide empirical evidence to aid in addressing policy and health disparities. Specifically, we conducted a 14-year time-trend analysis (2006–2019) of biennial age-standardized AM rates by sex and region using data from the Italian Institute of Statistics. Our objectives are to identify the extent of AM in Italy, examine regional and sex differences in AM, TM and PM rates, and explore the potential implications of these findings for public health policy and practice.

## 2 Methods

### 2.1 Study design

This time-trend analysis investigates the region- and sex-specific trends of AM in Italy. We used the joint OECD/Eurostat list [15] to calculate biennial age-standardized rates by sex and region.

### 2.2 Data sources

Mortality and population data from 2006 to 2019 were provided by the Italian Institute of Statistics. For mortality data, the Italian Institute of Statistics collects data through the death certificates of all citizens resident in Italy and consequently listed in the population registry.

The joint OECD/Eurostat list's ICD-10 codes related to AM, TM and PM were then extracted. AM is the sum of TM (i.e., deaths treatable by ideally functioning healthcare services) and PM (i.e., deaths preventable by preventive measures such as vaccines). According to the OECD guiding principles, in cases where there is no strong evidence of predominance in listing a death in one of the two categories (preventable and treatable), a 50%–50 % split was used. In the total (avoidable), any double counting of the same causes of death across the two lists was avoided [[15], [16], [17]].

### 2.3 Analysis

AM rate was calculated as the two-year average annual number of deaths over the mid-period population aged 0–74 years per 100,000 inhabitants, and was then stratified by age group, sex, and region (19 regions plus the two autonomous provinces (APs) of Trento and Bolzano). Causes of death classified as treatable or preventable were identified using the OECD/Eurostat lists of preventable and treatable causes of death. We computed age-standardized mortality rates using the European Standard Population revised in 2013 by the European Commission for the EU-27 and European Free Trade Association (EFTA).

## 3 Results

### 3.1 Avoidable mortality

Overall, AM showed a large reduction from 2006/7 to 2018/9 (from 221.0 to 166.4 per 100,000 pop) both overall (Table 1) and across Italian regions (Fig. S1).

**Table 1:** Overall Avoidable, Treatable and Preventable mortality rates (absolute values and deaths per 100,000 population).

Years	Avoidable		Treatable		Preventable	
	deaths	deaths per 100,000 pop	deaths	deaths per 100,000 pop	deaths	deaths per 100,000 pop
2006/7	235,143	221.0	86,913	81.9	148,230	139.0
2008/9	228,437	210.5	85,105	78.6	143,332	131.9
2010/1	219,020	199.0	82,350	74.9	136,670	124.1
2012/3	212,772	191.5	80,921	72.9	131,851	118.6
2014/5	206,117	182.4	78,713	69.8	127,404	112.6
2016/7	200,148	173.4	75,677	65.7	124,471	107.7
2018/9	194,550	166.4	74,894	64.1	119,656	102.2

**Fig. 1:**
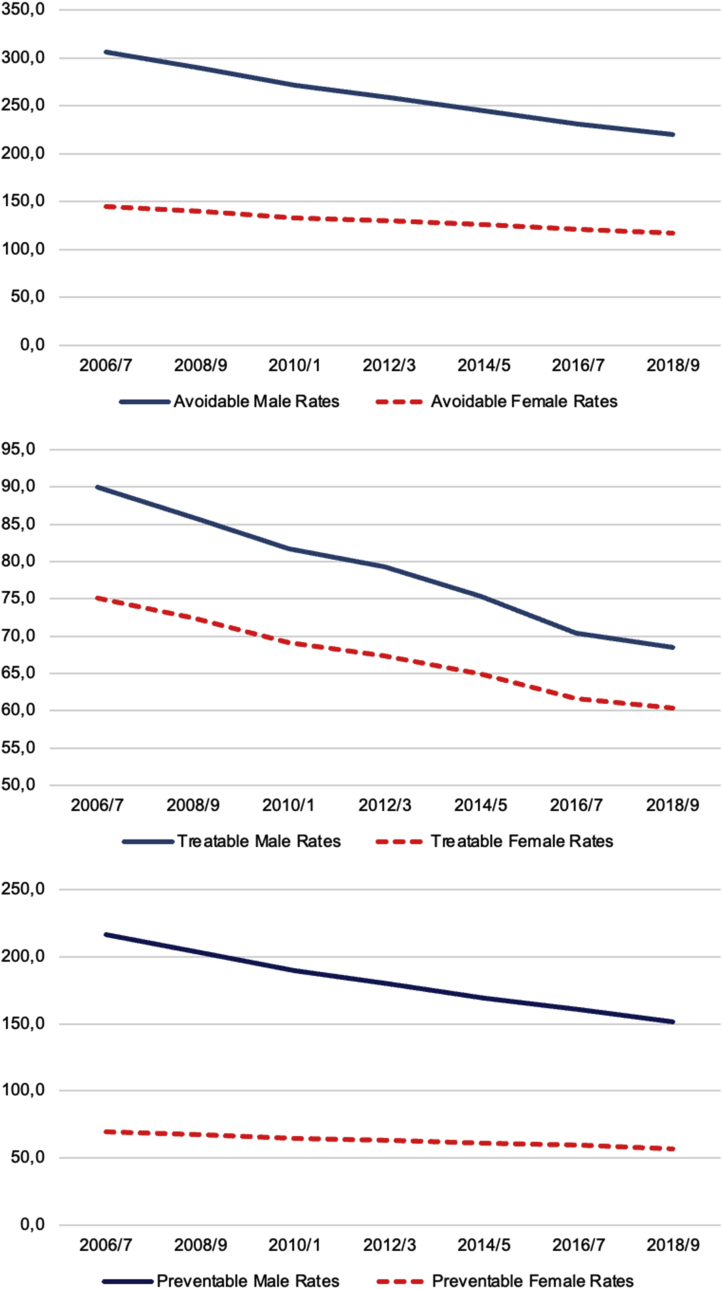
2006–2019 trend of overall (male plus female) Avoidable, Treatable and Preventable mortality in Italy.

**Fig. 2:**
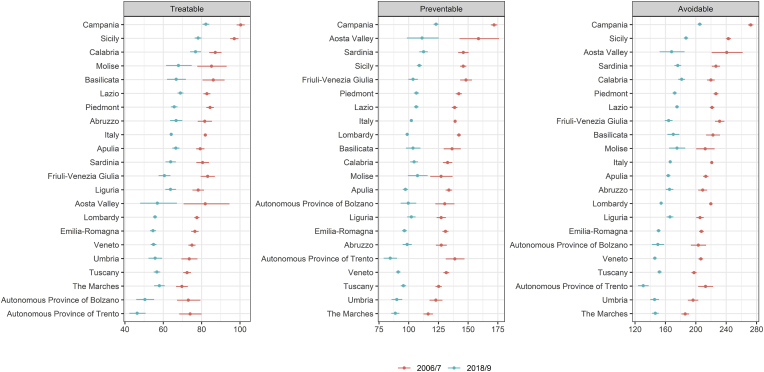
Regional and sex differences in Avoidable, Treatable and Preventable mortality in Italy between 2006/7 and 2018/9.

Remarkable sex differences were registered throughout the study period, with females showing lower rates of AM than males in both 2006/7 (144.5 vs. 306.3) and 2018/9 (117.1 vs. 219.9) (Table 2). Between 2006/7 and 2018/9 the % reduction in AM was −28.2 % in males and −19.0 % in females. However, sex differences in 2018/9 were lower than in 2006/7 (Fig. 1, Fig. 2).

**Table 2:** Regional values of Avoidable, Preventable and Treatable mortality rates by sex.

REGION	Rates 2006/7						Rates 2018/9						% difference 2006/7–2018/9					
	Male			Female			Male			Female			Male			Female		
	Avoid	Prev	Trea	Avoid	Prev	Trea	Avoid	Prev	Trea	Avoid	Prev	Trea	Avoid	Prev	Trea	Avoid	Prev	Trea
Piedmont	313,5	220,1	93,4	147,2	70,4	76,8	228,4	157,7	70,7	119,8	58,6	61,2	−27,1	−28,4	−24,2	−18,6	−16,8	−20,32
Aosta Valley	**332,1**	**246,1**	86,0	154,1	75,9	78,3	229,8	166,6	63,3	108,4	57,6	50,8	−30,8	−32,3	−26,4	**−29,7**	−24,1	**−35,1**
Lombardy	308,6	223,8	84,9	140,3	68,8	71,5	203,9	145,1	58,9	108,6	55,5	53,0	**−33,9**	**−35,2**	−30,6	−22,6	−19,3	−25,9
A.P. of Bolzano	284,9	199,3	85,6	**127,5**	65,9	**61,6**	207,2	151,4	**55,8**	**96,9**	51,1	**45,9**	−27,3	**−24,1**	**−34,8**	−24,0	−22,5	−25,6
A.P. of Trento	302,7	221,3	81,4	129,7	62,5	67,2	**177,7**	**127,9**	**49,7**	**85,3**	**42,2**	**43,1**	**−41,3**	**−42,2**	**−39,0**	**−34,2**	**−32,5**	**−35,9**
Veneto	290,3	206,0	84,3	130,5	63,6	**66,9**	194,8	135,1	59,7	**100,1**	49,4	**50,7**	**−32,9**	**−34,4**	−29,2	−23,3	−22,2	−24,3
Friuli-Venezia Giulia	310,5	221,8	88,7	**159,5**	**80,8**	**78,7**	219,1	154,7	64,4	113,1	55,9	57,3	−29,4	−30,2	−27,4	**−29,0**	**−30,9**	**−27,2**
Liguria	283,4	199,3	84,1	138,2	64,5	73,7	218,5	150,5	68,1	118,7	58,5	60,2	**−22,9**	−24,5	−19,0	−14,1	**−9,3**	−18,4
Emilia-Romagna	284,5	199,6	84,8	137,4	67,8	69,5	194,6	137,5	**57,1**	110,8	58,4	52,4	−31,6	−31,1	**−32,7**	−19,3	−13,9	−24,6
Tuscany	**274,6**	**196,1**	**78,5**	**128,6**	61,2	67,4	199,0	140,8	58,2	109,7	54,1	55,5	−27,6	−28,2	−25,9	−14,7	−11,5	−17,6
Umbria	**268,3**	**188,3**	**80,1**	131,1	63,0	68,1	**189,5**	**129,5**	59,9	105,7	53,6	52,0	−29,4	−31,2	−25,1	−19,4	−14,9	−23,6
Marche	**264,0**	**183,0**	**81,0**	**115,3**	**55,4**	**59,9**	**192,9**	**131,3**	61,6	103,8	**49,0**	54,8	−26,9	−28,3	−24,0	**−10,0**	**−11,5**	**−8,6**
Latium	309,1	216,3	92,9	144,9	70,7	74,2	230,78	154,8	76,0	**126,0**	**63,1**	62,9	−25,5	−28,4	−18,2	**−13,0**	**−10,7**	−15,3
Abruzzo	296,8	201,0	95,7	128,1	**59,4**	68,6	222,8	150,6	72,2	111,9	50,2	61,7	−24,9	−25,1	−24,6	**−12,7**	−15,5	**−10,3**
Molise	298,6	201,9	96,7	133,3	**58,4**	74,9	**246,7**	**169,2**	77,5	106,4	**47,6**	58,9	**−17,4**	**−16,2**	−19,8	−20,1	−18,5	−21,4
Campania	**370,3**	**260,8**	**109,5**	**185,6**	**93,0**	**92,6**	**268,6**	**180,6**	**88,0**	**147,4**	**70,4**	**77,1**	−27,5	−30,7	−19,7	−20,6	**−24,3**	−16,8
Apulia	292,6	210,5	82,1	142,4	65,4	77,1	216,9	148,6	68,3	115,2	50,1	65,1	−25,9	−29,4	**−16,8**	−19,1	−23,3	−15,6
Basilicata	312,9	217,0	95,9	140,5	63,3	77,2	228,4	156,7	71,7	115,2	53,1	62,1	−27,0	−27,8	−25,2	−18,0	−16,2	−19,5
Calabria	304,6	205,9	**98,7**	141,4	64,8	76,6	245,4	160,1	**85,3**	120,9	52,1	**68,9**	**−19,5**	**−22,3**	**−13,6**	−14,5	−19,6	**−10,1**
Sicily	326,7	223,6	**103,1**	**169,1**	**77,0**	**92,0**	**245,9**	162,7	**83,2**	**133,7**	**60,0**	**73,7**	−24,7	−27,2	**−19,3**	−20,9	−22,2	−19,9
Sardinia	**328,4**	**238,8**	89,6	132,8	60,3	72,5	245,6	**177,3**	68,3	110,6	50,8	59,8	−25,2	−25,7	−23,8	−16,7	−15,7	−17,6
**Italy**	306,3	216,2	90,0	144,5	69,3	75,1	219,9	151,4	68,5	117,1	56,7	60,3	−28,2	−30,0	−23,9	−19,0	−18,2	−19,7

**Fig. 3:**
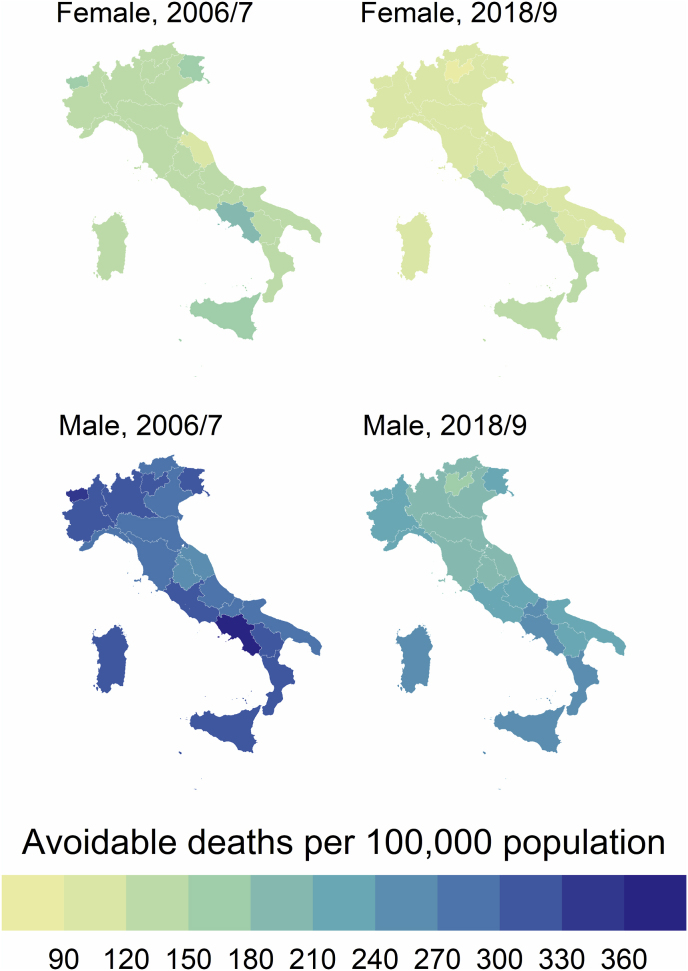
Sex differences in Avoidable mortality across Italian regions in 2006/7 and 2018/9.

Sex differences in AM were also registered across regions, both overall and in terms of % reduction between 2006/7 and 2018/9 (Table 2). We also registered a north–south gradient in AM, both in 2006/7 and 2018/9, with southern regions showing worse performance with higher values of AM in both time periods (Fig. 2). Sex differences in AM across regions also had a clear north–south gradient, with southern regions showing worse performance with higher sex differences in 2018/9 (Fig. 3). In 2006/7, the higher rates of AM for males were registered in Campania (370.3 per 100,000 population) and Aosta Valley (332.1), while for females in Campania (185.6) and Sicily (169.1) (Table 2). In 2018/9 the higher rates of AM for males were registered in Campania (268.6) and Molise (246.7), while for females in Campania (147.4) and Sicily (133.7). The highest % difference in AM between 2006/7 and 2018/9 both for males and females was found in the AP of Trento (−41.3 % and −34.2 %, respectively), while the lowest reduction was found in Molise for males (−17.4 %) and in Marche for females (−10.0 %) (Table 2).

### 3.2 Treatable mortality

Overall, TM showed a large reduction from 2006/7 to 2018/9 (from 81.9 to 64.1 per 100,000 population) both overall (Table 1) and across Italian regions (Fig. S1). The decreasing trend of TM was more pronounced than that of AM and PM (Fig. 1).

Remarkable sex differences were registered throughout the study period, with females showing lower rates than males in TM in both 2006/7 (75.1 vs. 90.0) and 2018/9 (60.3 vs. 68.5) (Table 2). Between 2006/7 and 2018/9 the % reduction in TM was −23.9 % in males and −19.7 % in females. However, sex gaps in 2018/9 were lower than in 2006/7 (Figs. 1 and 2).

**Fig. 4:**
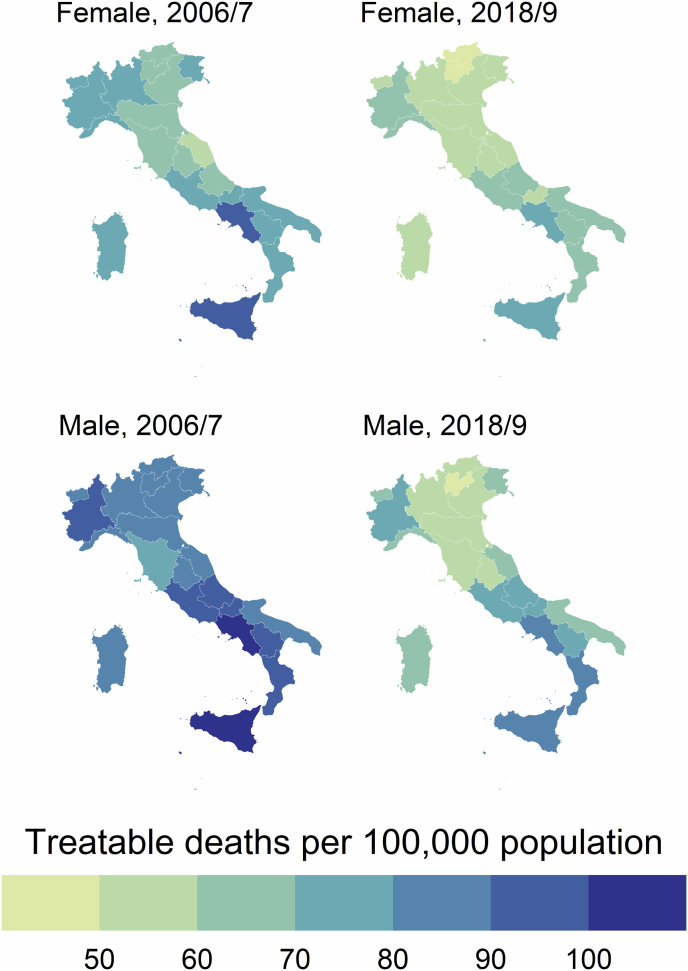
Sex differences in Treatable mortality across Italian regions in 2006/7 and 2018/9.

Sex differences in TM were also registered across regions, both overall and in terms of % reduction between 2006/7 and 2018/9 (Table 2). We also registered a north–south gradient in TM, both in 2006/7 and 2018/9, with southern regions showing worse performance with higher values of TM in both time periods (Fig. 2). Sex differences in TM across regions also had a clear north–south gradient, with southern regions showing worse performance with higher sex differences in 2018/9 (Fig. 4). In 2006/7, the highest rates of TM for males were registered in Campania (109.5 per 100,000 population) followed by Sicily (103.1), while for females in Campania (92.6) and Sicily (92.0) (Table 2). In 2018/9 the highest rates of TM for males were registered in Campania (88.0) and Calabria (85.3), while for females in Campania (77.1) and Sicily (73.7). The highest % difference in TM between 2006/7 and 2018/9 both for males and females was found in the AP of Trento (−39.0 % and −35.9 %, respectively), while the lower reduction was found in Calabria for males (−13.6 %) and in Marche for females (−8.6 %) (Table 2).

### 3.3 Preventable mortality

Overall, PM showed a large reduction from 2006/7 to 2018/9 (from 139.0 to 102.2 per 100,000 population) both overall (Table 1) and across Italian regions (Fig. S1).

Remarkable sex differences were registered throughout the study period, with females showing lower rates than males in both 2006/7 (216.2 vs. 69.3) and 2018/9 (151.4 vs. 56.7) (Table 2). Between 2006/7 and 2018/9 the % reduction in PM was −30.0 % in males and −18.2 % in females. However, sex gaps in 2018/9 were lower than in 2006/7 (Figs. 1 and 2).

**Fig. 5:**
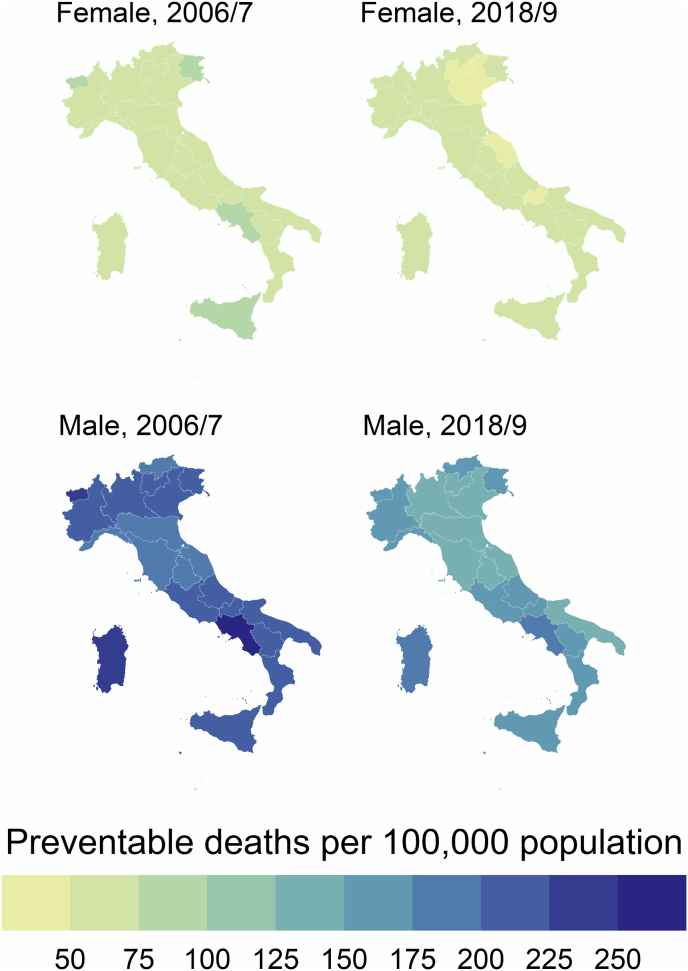
Sex differences in Preventable mortality across Italian regions in 2006/7 and 2018/9.

Sex differences in PM were also registered across regions, both overall and in terms of % reduction between 2006/7 and 2018/9 (Table 2). We also registered a north–south gradient in PM, both in 2006/7 and 2018/9, with southern regions showing worse performance with higher values of PM in both time periods (Fig. 2). Sex differences in PM across regions also had a clear north–south gradient, with southern regions showing worse performance with higher sex differences in 2018/9 (Fig. 5). In 2006/7, the highest rates of PM for males were registered in Campania (260.8 per 100,000 population) and Sardinia (238.8), while for females in Campania (93.0) and Friuli-Venezia Giulia (80.8) (Table 2). In 2018/9 the highest rates of PM for males were registered in Campania (180.6) and Sardinia (177.3), while for females in Campania (70.4) and Latium (63.1). The highest % difference in PM between 2006/7 and 2018/9 both for males and females was found in the AP of Trento (−42.2 % and −32.5 %, respectively), while the lowest reduction was found in Molise for males (−16.2 %) and in Liguria for females (−9.3 %) (Table 2).

## 4 Discussion

This study provides a comprehensive analysis of AM, including both TM and PM across region- and sex-specific domains within Italy. Our analysis spans a 14-year time period, from 2006 to 2019, offering critical insights into the patterns and disparities in AM, TM, and PM within the context of Italy's healthcare system.

From 2006/7 to 2018/9, a large reduction was observed in overall AM rates. Sex disparities were noted, with females consistently exhibiting lower AM rates than males. Moreover, these sex differences, as well as the north–south gradient of AM across regions, decreased over time, and the regions showing the highest AM rates varied across the study period.

Overall, TM rates exhibited a large reduction, with a more pronounced downward trend than AM and PM. Similarly to AM, sex disparities in TM were noted, although the sex gap decreased over time. A north–south gradient in TM rates across regions was also identified, with Southern regions showing higher TM rates. This gradient was also noted in sex disparities across regions.

A large reduction was also noted in overall PM rates. Similarly to AM and TM, sex disparities were identified, with females exhibiting lower PM rates. The sex gap and north–south gradient in PM across regions decreased over time, mirroring the trends in AM and TM.

In line with prior research [12,13,[15], [16], [17]], our findings present compelling evidence of a north–south gradient in AM, PM, and TM rates in Italy, observable both in 2006/7 and 2018/9. Southern regions consistently demonstrated poorer performance with elevated mortality rates during these time periods, compared to northern regions (with some exceptions). This north–south divide reflects an enduring challenge for Italy's healthcare system, mirroring disparities in socioeconomic status, health infrastructure, and access to quality healthcare services between these areas. The persistence of this gradient over time underscores the necessity for sustained, targeted interventions to address regional disparities and improve health outcomes, particularly in southern Italy. From a sex perspective, our data uncovers remarkable disparities as well. In 2006/7, sex differences in AM, PM, and TM rates were much higher compared to the 2018/9 data. While this suggests that some progress has been made towards sex parity, regional disparities in this respect are still profound and exhibit considerable variability. Southern regions fared worse in terms of sex equality, exhibiting higher rates of sex-related AM. This observation suggests the need for sex-responsive health policies and interventions, particularly in regions with poorer performance. Indeed, the registered sex gap and regional disparities could be attributed to various factors, including demographic shifts, changing disease patterns, and alterations in healthcare funding [12,13,[15], [16], [17], [18], [19]]. In particular, the growing prevalence of chronic diseases, such as cardiovascular disease, cancer, and respiratory diseases, likely due to an aging population, appears to be driving an increase in AM rates among men [20,21].

Public healthcare funding cuts, which plenty of literature suggests being inversely correlated with mortality, may also be exacerbating sex differences in AM. Austerity measures implemented over the past decade have reduced funding for public healthcare services, leading to decreased quality and availability of healthcare [22]. In the light of our results (large improvement in AM), this apparent paradox can be attributed to a combination of factors, including increased efficiency in healthcare delivery, targeted public health interventions, and advancements in medical technology. These positive outcomes reflect Italy's commitment to optimizing healthcare resource allocation and continuously enhancing its healthcare system's performance despite financial constraint [23]. Accordingly, to tackle sex differences in AM, it is crucial to preserve adequate funds for the healthcare system and establish policies and interventions addressing the social, cultural, and biological factors contributing to these disparities.

A noteworthy aspect of our study is the relatively low values of AM, PM, and TM, given the maturity of Italy's healthcare system as one characteristic of a high-income country. Despite encouraging, this finding raises an important point about the limitations of further reductions in AM and PM, which cannot feasibly go beyond a certain intrinsic threshold inherent in every health system. As such, strategies for reducing AM should also prioritize the equitable distribution of health outcomes and resources across sex and regional domains, rather than a narrow focus on overall reductions.

Aligned with the wider body of research [24,25], our findings confirm the utility of the AM concept as a robust measure of health system performance.

Possibly, funding the public sector appears to be more successful in reducing amenable deaths than a similar investment in private provision in Italy [26,27]. Within this context, the European PNRR (Recovery Plan) and Next Generation EU programs may play a crucial role in the enhancement of healthcare in Southern Italy, aimed at minimizing the disparity with Northern regions. Their funding could facilitate the development of medical infrastructure and access to advanced healthcare services, fostering regional equality. This will not only improve patient outcomes in the south, but also promote a more balanced, equitable healthcare landscape across Italy as a whole.

Our study contributes to the body of literature by shedding light on the intricate dynamics of AM, PM, and TM in Italy, both from a temporal and spatial/geographical perspective. Our study thus reinforces the evidence base for policy initiatives aimed at reducing health disparities and enhancing health system performance.

## 5 Strengths and limitations of the study

Given our use of aggregated data, we were unable to examine individual-level factors that may influence AM rates. These factors, such as individual health behaviors, genetic predispositions, and comorbidities, could provide a more nuanced understanding of AM and should be considered in future research. Additionally, our analysis covers data up to 2019 only, the latest available due to publication latency, which does not reflect accurately the most recent trends. Changes in healthcare delivery, public health policies, and societal norms post-2019 could impact AM rates and should be captured in ongoing surveillance efforts.

Despite these limitations, our study holds considerable value for public health decision-makers. By presenting a detailed analysis of AM patterns and disparities, our research informs the design and implementation of public health strategies.

Our findings underscore the need for regionally tailored, sex-responsive interventions that account for the diverse factors influencing health outcomes. Additionally, our study brings into focus the importance of continuous monitoring and evaluation of health system performance using measures like AM.

## 6 Conclusions

In conclusion, our study sheds light on the patterns and disparities in AM in Italy. The notable regional and sex disparities, as well as the persistent north–south gradient in health outcomes, call for targeted interventions to address these gaps. As Italy strives to improve its health system performance quality and achieve health equity, monitoring and addressing AM remain critical not only to save lives, but also to ensure that health resources are allocated effectively, that inequalities are reduced and that the general health and well-being of the population is improved. This requires the adoption of innovative approaches based on data and evidence.

## Ethical approval

This research required no ethical approval as it is not a research on human subjects.

## Funding

This research received no external founding.

## Data availability

The data that support the findings of this study are available on request from the corresponding author. The data are not publicly available due to privacy or ethical restrictions.

## Declaration of competing interest

The authors declare that they have no known competing financial interests or personal relationships that could have appeared to influence the work reported in this paper.

## Figures and Tables

**Fig. S1:**