# Supplementary material for: Regional and sex inequalities of avoidable mortality in Italy: A time trend analysis
Source: Public Health Pract (Oxf). 2023 Oct 26;6:100449. doi: 10.1016/j.puhip.2023.100449 (PMC10643453; doi:10.1016/j.puhip.2023.100449)
Supplement: Multimedia component 1 [file mmc1.pdf]

REGION	Rates 2006-2007						2006-2007 lower limit						2006-2007 upper limit						Rates 2018-2019						2018-2019 lower limit						2018-2019 upper limit					
	TR M	AV M	PR M	TR F	AV F	PR F	TR M	AV M	PR M	TR F	AV F	PR F	TR M	AV M	PR M	TR F	AV F	PR F	TR M	AV M	PR M	TR F	AV F	PR F	TR M	AV M	PR M	TR F	AV F	PR F	TR M	AV M	PR M	TR F	AV F	PR F
Piedimont	93,4	313,5	220,1	76,8	147,2	70,4	90,4	308,1	215,6	74,2	143,6	67,9	96,4	319,0	224,7	79,4	150,8	72,9	70,7	228,4	157,7	61,2	119,8	58,6	68,2	223,9	153,9	59,0	116,6	56,4	73,3	233,0	161,5	63,6	123,1	60,9
Aosta Valley	86,0	332,1	246,1	78,3	154,1	75,9	69,5	299,0	217,7	63,2	132,6	60,9	105,2	368,0	277,2	96,0	178,3	93,5	63,3	229,8	166,6	50,8	108,4	57,6	50,0	203,7	144,4	39,2	91,1	45,2	79,1	258,6	191,3	65,0	128,2	72,5
Lombardy	84,9	308,6	223,8	71,5	140,3	68,8	82,9	304,9	220,6	69,8	137,9	67,1	86,9	312,4	227,0	73,3	142,8	70,5	58,9	203,9	145,1	53,0	108,6	55,5	57,3	201,0	142,6	51,6	106,5	54,1	60,5	206,9	147,5	54,5	110,6	57,0
AP of Bolzano	85,6	284,9	199,3	61,6	127,5	65,9	76,5	268,1	185,4	54,2	116,7	58,2	95,5	302,4	214,0	69,7	138,9	74,2	55,8	207,2	151,4	45,9	96,9	51,1	49,0	194,0	140,1	40,0	88,3	44,8	63,3	221,1	163,3	52,4	106,2	57,9
AP of Trento	81,4	302,7	221,3	67,2	129,7	62,5	72,9	286,2	207,2	59,9	119,4	55,4	90,7	320,0	236,1	75,3	140,7	70,2	49,7	177,7	127,9	43,1	85,3	42,2	43,8	166,2	118,3	37,7	77,6	36,8	56,3	189,7	138,2	49,2	93,6	48,1
Veneto	84,3	290,3	206,0	66,9	130,5	63,6	81,5	285,1	201,6	64,6	127,2	61,2	87,2	295,7	210,5	69,4	133,9	66,0	59,7	194,8	135,1	50,7	100,1	49,4	57,5	190,8	131,8	48,7	97,3	47,5	62,0	198,9	138,5	52,7	103,0	51,5
Friuli-Venezia Giulia	88,7	310,5	221,8	78,7	159,5	80,8	83,3	300,3	213,2	73,8	152,5	75,9	94,4	321,0	230,7	83,8	166,7	86,0	64,4	219,1	154,7	57,3	113,1	55,8	59,9	210,8	147,8	53,2	107,4	51,8	69,1	227,7	162,0	61,7	119,1	60,1
Liguria	84,1	283,4	199,3	73,7	138,2	64,5	79,6	275,1	192,4	69,7	132,8	60,8	88,8	291,9	206,5	77,9	143,9	68,4	68,1	218,5	150,5	60,2	118,7	58,5	64,0	211,2	144,4	56,5	113,5	55,0	72,3	226,1	156,7	64,0	124,1	62,3
Emilia-Romagna	84,8	284,5	199,6	69,5	137,4	67,8	81,9	279,1	195,2	67,0	133,8	65,4	87,8	289,9	204,2	72,1	141,0	70,4	57,1	194,6	137,5	52,4	110,8	58,4	54,8	190,4	134,0	50,3	107,8	56,2	59,5	198,9	141,2	54,6	113,9	60,7
Toscany	78,5	274,6	196,1	67,4	128,6	61,2	75,5	269,0	191,4	64,8	124,9	58,7	81,6	280,3	200,9	70,1	132,3	63,8	58,2	199,0	140,8	55,5	109,7	54,1	55,7	194,4	136,9	53,2	106,4	51,8	60,8	203,6	144,7	57,9	113,0	56,5
Umbria	80,1	268,3	188,3	68,1	131,1	63,0	73,9	257,0	178,8	62,7	123,6	57,8	86,6	280,0	198,1	73,9	139,1	68,6	59,9	189,5	129,5	52,0	105,7	53,6	54,8	180,3	121,9	47,4	99,1	49,0	65,5	199,1	137,5	57,0	112,6	58,6
Marche	81,0	264,0	183,0	59,9	115,3	55,4	76,3	255,5	175,9	56,0	109,9	51,7	85,9	272,8	190,4	64,0	120,9	59,3	61,6	192,9	131,3	54,8	103,8	49,0	57,6	185,8	125,5	51,1	98,8	45,6	65,8	200,2	137,4	58,6	109,0	52,7
Latium	92,9	309,1	216,3	74,2	144,9	70,7	90,1	304,1	212,0	71,9	141,7	68,4	95,7	314,3	220,6	76,6	148,2	73,0	76,0	230,8	154,8	62,9	126,0	63,1	73,6	226,7	151,4	60,9	123,2	61,1	78,4	234,9	158,2	65,0	129,0	65,2
Abruzzo	95,7	296,8	201,0	68,8	128,1	59,4	90,1	286,8	192,9	64,2	121,9	55,2	101,7	307,0	209,5	73,6	134,7	63,9	72,2	222,8	150,6	61,7	111,9	50,2	67,5	214,5	143,9	57,5	106,3	46,5	77,1	231,2	157,6	66,1	117,7	54,2
Molise	96,7	298,6	201,9	74,9	133,3	58,4	85,3	278,4	185,4	65,4	120,5	50,0	109,0	319,8	219,6	85,4	147,1	67,8	77,5	246,7	169,2	58,9	106,4	47,6	67,8	229,2	154,8	50,6	95,2	40,2	88,2	265,2	184,6	68,2	118,7	56,0
Campania	109,5	370,3	260,8	92,6	185,6	93,0	106,4	364,4	255,9	89,9	181,7	90,2	112,8	376,2	265,7	95,4	189,5	95,8	88,0	268,6	180,6	77,1	147,4	70,4	85,4	264,1	176,9	74,7	144,2	68,2	90,7	273,2	184,4	79,4	150,7	72,6
Apulia	82,1	292,6	210,5	77,1	142,4	65,4	79,0	286,7	205,5	74,2	138,6	62,8	85,3	298,6	215,6	80,0	146,4	68,1	68,3	216,9	148,6	65,1	115,2	50,1	65,7	212,2	144,7	62,6	111,9	48,0	71,1	221,7	152,6	67,6	118,5	52,3
Basilicata	95,9	312,9	217,0	77,2	140,5	63,3	87,3	297,4	204,1	69,9	130,7	56,8	105,0	329,0	230,4	84,9	150,8	70,4	71,7	228,4	156,7	62,1	115,2	53,1	64,6	215,7	146,2	55,7	106,4	47,2	79,4	241,8	167,8	69,1	124,5	59,5
Calabria	98,7	304,6	205,9	76,6	141,4	64,8	93,9	296,1	199,0	72,5	135,9	61,1	103,7	313,3	213,0	80,9	147,1	68,7	85,3	245,4	160,1	68,9	120,9	52,1	81,1	238,1	154,2	65,1	116,0	48,9	89,7	252,8	166,0	72,7	126,0	55,4
Sicily	103,1	326,7	223,6	92,0	169,1	77,0	99,9	321,1	219,0	89,2	165,2	74,5	106,3	332,4	228,4	94,9	172,9	79,6	83,2	245,9	162,7	73,7	133,7	60,0	80,5	241,3	159,0	71,3	130,5	57,8	85,9	250,5	166,5	76,2	137,0	62,2
Sardinia	89,6	328,4	238,8	72,5	132,8	60,3	84,7	319,0	230,8	68,3	127,1	56,5	94,8	338,0	247,0	76,9	138,7	64,3	68,3	245,6	177,3	59,8	110,6	50,8	64,3	238,1	170,9	56,2	105,7	47,5	72,5	253,4	183,9	63,6	115,7	54,3
Italy	90,0	306,3	216,2	75,1	144,5	69,3	89,2	304,7	215,0	74,4	143,5	68,7	90,9	307,8	217,5	75,9	145,5	70,0	68,5	219,9	151,4	60,3	117,1	56,7	67,8	218,6	150,4	59,7	116,2	56,1	69,2	221,1	152,4	61,0	117,9	57,3

REGION	Difference 2006/7 - 2018/9 in absolute values						Difference in absolute values lower limit						Difference in absolute values upper limit						Difference 2006/7 - 2018/9 in %						Difference in % lower limit						Difference in % upper limit					
	TR M	AV M	PR M	TR F	AV F	PR F	TR M	AV M	PR M	TR F	AV F	PR F	TR M	AV M	PR M	TR F	AV F	PR F	TR M	AV M	PR M	TR F	AV F	PR F	TR M	AV M	PR M	TR F	AV F	PR F	TR M	AV M	PR M	TR F	AV F	PR F
Piedimont	-22,6	-85,1	-62,5	-15,6	-27,4	-11,8	-22,2	-84,2	-61,7	-15,3	-27,0	-11,6	-23,0	-86,0	-63,2	-15,9	-27,8	-12,1	-24,2	-27,1	-28,4	-20,3	-18,6	-16,8	-24,5	-27,3	-28,6	-20,6	-18,8	-17,0	-23,9	-27,0	-28,1	-20,0	-18,4	-16,5
Aosta Valley	-22,7	-102,3	-79,6	-27,5	-45,8	-18,3	-19,5	-95,3	-73,4	-24,0	-41,5	-15,7	-26,0	-109,4	-85,9	-31,0	-50,2	-21,0	-26,4	-30,8	-32,3	-35,1	-29,7	-24,1	-28,1	-31,9	-33,7	-38,0	-31,3	-25,8	-24,8	-29,7	-31,0	-32,3	-28,1	-22,4
Lombardy	-26,0	-104,7	-78,7	-18,5	-31,8	-13,3	-25,6	-103,8	-78,0	-18,2	-31,4	-13,0	-26,4	-105,6	-79,5	-18,8	-32,1	-13,5	-30,6	-33,9	-35,2	-25,9	-22,6	-19,3	-30,9	-34,1	-35,4	-26,1	-22,8	-19,4	-30,4	-33,8	-35,0	-25,7	-22,5	-19,1
AP of Bolzano	-29,8	-77,7	-47,9	-15,8	-30,6	-14,8	-27,5	-74,2	-45,2	-14,2	-28,5	-13,4	-32,2	-81,3	-50,7	-17,3	-32,7	-16,3	-34,8	-27,3	-24,1	-25,6	-24,0	-22,5	-35,9	-27,7	-24,4	-26,3	-24,4	-23,0	-33,7	-26,9	-23,7	-24,9	-23,6	-22,0
AP of Trento	-31,7	-125,1	-93,4	-24,1	-44,4	-20,3	-29,1	-119,9	-88,9	-22,2	-41,8	-18,6	-34,4	-130,4	-97,9	-26,1	-47,1	-22,1	-39,0	-41,3	-42,2	-35,9	-34,2	-32,5	-40,0	-41,9	-42,9	-37,0	-35,0	-33,5	-37,9	-40,7	-41,5	-34,7	-33,5	-31,4
Veneto	-24,7	-95,5	-70,9	-16,3	-30,4	-14,1	-24,1	-94,3	-69,8	-15,9	-29,8	-13,7	-25,3	-96,8	-72,0	-16,7	-31,0	-14,5	-29,2	-32,9	-34,4	-24,3	-23,3	-22,2	-29,5	-33,1	-34,6	-24,6	-23,5	-22,4	-29,0	-32,7	-34,2	-24,0	-23,1	-22,0
Friuli-Venezia Giulia	-24,3	-91,4	-67,1	-21,4	-46,3	-24,9	-23,3	-89,5	-65,4	-20,6	-45,1	-24,0	-25,3	-93,3	-68,7	-22,2	-47,5	-25,9	-27,4	-29,4	-30,2	-27,2	-29,0	-30,9	-28,0	-29,8	-30,7	-27,9	-29,6	-31,7	-26,8	-29,1	-29,8	-26,4	-28,5	-30,1
Liguria	-16,0	-64,9	-48,9	-13,5	-19,5	-6,0	-15,6	-63,9	-48,0	-13,2	-19,2	-5,9	-16,4	-65,9	-49,7	-13,8	-19,8	-6,1	-19,0	-22,9	-24,5	-18,4	-14,1	-9,3	-19,6	-23,2	-25,0	-19,0	-14,5	-9,7	-18,5	-22,6	-24,1	-17,8	-13,8	-8,9
Emilia-Romagna	-27,7	-89,8	-62,1	-17,1	-26,5	-9,5	-27,1	-88,7	-61,2	-16,6	-26,0	-9,2	-28,4	-90,9	-63,0	-17,5	-27,0	-9,7	-32,7	-31,6	-31,1	-24,6	-19,3	-13,9	-33,1	-31,8	-31,4	-24,8	-19,5	-14,0	-32,3	-31,4	-30,9	-24,3	-19,2	-13,8
Toscany	-20,3	-75,7	-55,3	-11,9	-18,9	-7,1	-19,8	-74,6	-54,5	-11,6	-18,6	-6,8	-20,8	-76,7	-56,2	-12,2	-19,3	-7,3	-25,9	-27,6	-28,2	-17,6	-14,7	-11,5	-26,2	-27,8	-28,5	-17,9	-14,9	-11,7	-25,5	-27,4	-28,0	-17,4	-14,6	-11,4
Umbria	-20,1	-78,9	-58,7	-16,1	-25,5	-9,4	-19,2	-76,8	-56,9	-15,3	-24,5	-8,8	-21,1	-81,0	-60,6	-16,9	-26,5	-10,0	-25,1	-29,4	-31,2	-23,6	-19,4	-14,9	-25,9	-29,9	-31,8	-24,4	-19,8	-15,3	-24,4	-28,9	-30,6	-22,9	-19,0	-14,5
Marche	-19,4	-71,1	-51,7	-5,2	-11,5	-6,4	-18,7	-69,7	-50,5	-4,9	-11,1	-6,1	-20,2	-72,6	-53,0	-5,4	-11,9	-6,7	-24,0	-26,9	-28,3	-8,6	-10,0	-11,5	-24,5	-27,3	-28,7	-8,7	-10,1	-11,8	-23,4	-26,6	-27,8	-8,5	-9,9	-11,2
Latium	-16,9	-78,4	-61,5	-11,3	-18,9	-7,5	-16,5	-77,4	-60,6	-11,1	-18,5	-7,3	-17,3	-79,3	-62,4	-11,6	-19,2	-7,8	-18,2	-25,4	-28,4	-15,3	-13,0	-10,7	-18,3	-25,5	-28,6	-15,4	-13,1	-10,7	-18,1	-25,2	-28,3	-15,2	-13,0	-10,6
Abruzzo	-23,6	-74,0	-50,4	-7,1	-16,3	-9,2	-22,6	-72,3	-49,0	-6,7	-15,6	-8,7	-24,6	-75,8	-51,9	-7,5	-16,9	-9,7	-24,6	-24,9	-25,1	-10,3	-12,7	-15,5	-25,1	-25,2	-25,4	-10,4	-12,8	-15,7	-24,2	-24,7	-24,8	-10,2	-12,6	-15,2
Molise	-19,2	-51,9	-32,8	-16,0	-26,8	-10,8	-17,6	-49,2	-30,6	-14,8	-25,3	-9,8	-20,8	-54,7	-35,0	-17,2	-28,4	-11,8	-19,8	-17,4	-16,2	-21,4	-20,1	-18,5	-20,6	-17,7	-16,5	-22,6	-21,0	-19,6	-19,1	-17,1	-15,9	-20,1	-19,3	-17,4
Campania	-21,5	-101,7	-80,1	-15,5	-38,1	-22,6	-21,0	-100,4	-78,9	-15,1	-37,5	-22,1	-22,1	-103,0	-81,3	-15,9	-38,8	-23,2	-19,7	-27,5	-30,7	-16,8	-20,6	-24,3	-19,7	-27,5	-30,9	-16,8	-20,6	-24,5	-19,6	-27,4	-30,6	-16,7	-20,5	-24,2
Apulia	-13,8	-75,7	-61,9	-12,0	-27,3	-15,3	-13,3	-74,5	-60,8	-11,6	-26,7	-14,8	-14,3	-76,9	-63,0	-12,4	-27,8	-15,7	-16,8	-25,9	-29,4	-15,6	-19,1	-23,3	-16,8	-26,0	-29,6	-15,7	-19,3	-23,6	-16,7	-25,7	-29,2	-15,5	-19,0	-23,1
Basilicata	-24,2	-84,4	-60,3	-15,1	-25,3	-10,3	-22,7	-81,7	-57,9	-14,2	-24,2	-9,6	-25,6	-87,2	-62,7	-15,9	-26,4	-10,9	-25,2	-27,0	-27,8	-19,5	-18,0	-16,2	-26,0	-27,5	-28,4	-20,3	-18,5	-16,9	-24,4	-26,5	-27,2	-18,7	-17,5	-15,5
Calabria	-13,4	-59,2	-45,9	-7,8	-20,5	-12,7	-12,8	-58,0	-44,7	-7,4	-19,9	-12,2	-14,0	-60,5	-47,0	-8,1	-21,1	-13,2	-13,6	-19,5	-22,3	-10,1	-14,5	-19,6	-13,7	-19,6	-22,5	-10,2	-14,6	-20,0	-13,5	-19,3	-22,1	-10,0	-14,3	-19,3
Sicily	-19,9	-80,8	-60,9	-18,3	-35,4	-17,1	-19,4	-79,8	-60,0	-17,9	-34,8	-16,7	-20,4	-81,9	-61,9	-18,7	-36,0	-17,5	-19,3	-24,7	-27,2	-19,9	-20,9	-22,2	-19,4	-24,8	-27,4	-20,0	-21,0	-22,4	-19,2	-24,6	-27,1	-19,7	-20,8	-22,0
Sardinia	-21,3	-82,8	-61,5	-12,7	-22,2	-9,5	-20,3	-80,9	-59,9	-12,1	-21,4	-9,0	-22,3	-84,7	-63,1	-13,4	-23,0	-10,0	-23,8	-25,2	-25,7	-17,6	-16,7	-15,7	-24,0	-25,4	-25,9	-17,7	-16,8	-15,9	-23,5	-25,1	-25,5	-17,4	-16,6	-15,6
Italy	-21,6	-86,4	-64,8	-14,8	-27,4	-12,6	-21,4	-86,1	-64,6	-14,7	-27,3	-12,5	-21,7	-86,7	-65,1	-14,9	-27,5	-12,7	-23,9	-28,2	-30,0	-19,7	-19,0	-18,2	-24,0	-28,3	-30,0	-19,7	-19,0	-18,2	-23,9	-28,2	-29,9	-19,6	-18,9	-18,1

TR M: treatable mortality male **AV M:** avoidable mortality male **PR M:** preventable mortality male **TR F:** treatable mortality male **AV F:** avoidable mortality male **PR F:** preventable mortality male
